# Loss of Rare Fish Species from Tropical Floodplain Food Webs Affects Community Structure and Ecosystem Multifunctionality in a Mesocosm Experiment

**DOI:** 10.1371/journal.pone.0084568

**Published:** 2014-01-08

**Authors:** Richard M. Pendleton, David J. Hoeinghaus, Luiz C. Gomes, Angelo A. Agostinho

**Affiliations:** 1 Department of Biological Sciences and the Institute of Applied Sciences, University of North Texas, Denton, Texas, United States of America; 2 Departamento de Biologia e Núcleo de Pesquisas em Limnologia, Ictiologia e Aquicultura (Nupélia), Universidade Estadual de Maringá, Maringá, Paraná, Brasil; Leibniz Center for Tropical Marine Ecology, Germany

## Abstract

Experiments with realistic scenarios of species loss from multitrophic ecosystems may improve insight into how biodiversity affects ecosystem functioning. Using 1000 L mesocoms, we examined effects of nonrandom species loss on community structure and ecosystem functioning of experimental food webs based on multitrophic tropical floodplain lagoon ecosystems. Realistic biodiversity scenarios were developed based on long-term field surveys, and experimental assemblages replicated sequential loss of rare species which occurred across all trophic levels of these complex food webs. Response variables represented multiple components of ecosystem functioning, including nutrient cycling, primary and secondary production, organic matter accumulation and whole ecosystem metabolism. Species richness significantly affected ecosystem function, even after statistically controlling for potentially confounding factors such as total biomass and direct trophic interactions. Overall, loss of rare species was generally associated with lower nutrient concentrations, phytoplankton and zooplankton densities, and whole ecosystem metabolism when compared with more diverse assemblages. This pattern was also observed for overall ecosystem multifunctionality, a combined metric representing the ability of an ecosystem to simultaneously maintain multiple functions. One key exception was attributed to time-dependent effects of intraguild predation, which initially increased values for most ecosystem response variables, but resulted in decreases over time likely due to reduced nutrient remineralization by surviving predators. At the same time, loss of species did not result in strong trophic cascades, possibly a result of compensation and complexity of these multitrophic ecosystems along with a dominance of bottom-up effects. Our results indicate that although rare species may comprise minor components of communities, their loss can have profound ecosystem consequences across multiple trophic levels due to a combination of direct and indirect effects in diverse multitrophic ecosystems.

## Introduction

Humans have altered and transformed ecosystems more in the past 50 years than any time in human history, and global biodiversity is currently declining at a rate a thousand times faster than observed in fossil records [1]. Understanding specific ways that biodiversity loss affects ecosystem structure and function is a pressing concern and an area of rapid research growth [2]–[5]. Seminal biodiversity and ecosystem functioning (BEF) studies were primarily conducted in temperate, terrestrial ecosystems and focused on diversity within a single trophic level using randomly constructed assemblages [6]. In general, these studies suggest a positive relationship exists between biodiversity and ecosystem functioning, yet this relationship can vary among ecosystem types and ecosystem processes [2], [6]. However, some have criticized if these studies realistically portrayed natural assemblages and ecosystems [3], [4].

BEF studies may improve realism by incorporating abundance, evenness, and interactions among species in a manner similar to that observed in nature [5]. Despite a rapid growth in BEF research, few studies have experimentally manipulated diversity in multitrophic systems (∼7% of studies reviewed by [6]) even though natural ecosystems can be diverse within and among trophic levels (i.e. horizontal and vertical diversity, *sensu* [7]). Furthermore, as species richness and trophic diversity increases, so does the number of potential direct and indirect interactions [8]. As a result, biodiversity loss across multiple trophic levels likely has a greater impact on the overall functioning of an ecosystem than loss within a single trophic level [7], [9].

Recent empirical and modeling studies have incorporated realism by explicitly testing effects of nonrandom species loss on ecosystem structure and function [10], [11], [12]. Species identity and the order in which species are lost can lead to different effects on trophic interactions and community structure [10], [11], [13]. Species loss is often nonrandom and can be influenced by body size, trophic position, rarity, and tolerance to stressors [14], [15]. In particular, rarity (defined here as low abundance) can be a good predictor of extinction risk [14], [15].

Studies examining effects of loss of rare species on ecosystem functioning have yielded mixed results due in part to the relative contribution of rare species to the particular ecosystem process measured [11], [13], [16]. These previous studies evaluated a single ecosystem process, and therefore may not have been able to detect the realized contribution of rare species to ecosystem multifunctionality (i.e. the ability of an ecosystem to maintain multiple functions simultaneously). However, a recent study found loss of rare species strongly influenced multiple ecosystem parameters across trophic levels [17]. Expanding the number of processes measured may better depict the multifunctionality of an intact community and its influence on ecosystem structure and function over time [18]–[20].

The few studies cited above indicate there is promise in using natural multitrophic assemblages as the framework to experimentally test ecosystem responses of nonrandom species loss in a manner that can provide insight into community processes and ecosystem functioning [2], [5]. As this research area continues to develop, it is important to also consider specific ecosystem types that are currently under-represented in such research. Tropical and freshwater ecosystems are highly diverse, strongly affected by human activities, and increasingly imperiled [1], [21]. However, freshwater ecosystems to date have received little attention when compared to terrestrial ecosystems, and research examining effects of biodiversity loss on ecosystem functioning in tropical ecosystems is lacking [6].

Here we present findings from a mesocosm experiment that tested the influence of nonrandom species loss on community structure and ecosystem multifunctionality. Long-term field data from floodplain lagoons were used to identify realistic experimental assemblages. Treatments replicating sequential loss of rare fish species were established using nested subsets of species from a summed rank abundance curve generated from the observed abundances of all species across lagoons. Our diverse experimental ecosystems include complex multitrophic interactions among taxa (i.e. within fish assemblages) and across the aquatic community (e.g. fish, zooplankton, and phytoplankton), thereby providing insight into interactions within (competitive effects) and across (consumptive effects) trophic levels. This study builds on previous research by testing effects of realistic scenarios of nonrandom species loss in a complex multitrophic system, and expands the scope of such studies to species-rich and under-represented ecosystems of the Neotropics. The main objectives of this study were to (1) investigate the effect of sequential loss of rare fish species on community and ecosystem responses over time, and (2) identify direct trophic interactions within these experimental food webs by which loss of rare species affected ecosystem multifunctionality.

## Materials and Methods

### Ethics Statement

All species were properly collected and handled in an ethical manner and with all required permissions from the Brazilian Environmental Ministry (Ministério do Meio Ambiente (MMA), Instituto Chico Mendes de Conservação da Biodiversidade (ICMBio), Sistema de autorização e informação em Biodiversidade (SISBIO)) under protocol number 22442-1, authentication code: 3263346. No other permissions were required for completion of this research, and this study does not include endangered or otherwise protected species.

### Description of Study System and Foundation for the Experimental Design

This research was conducted at the Upper Paraná River Floodplain Long Term Ecological Research (LTER) program’s field station located at the Upper Paraná River floodplain, Brazil. The Paraná River is the tenth largest river in the world in annual discharge (5.0×10^8^ m^3^yr^−1^) and fourth in drainage area (2.8×10^6^ km^2^) [22]. The upper third of the Paraná River basin (891,000 km^2^) is contained almost completely within Brazil, including Brazil’s most densely populated region [22]. The Upper Paraná River basin is extensively impounded, with the last free-flowing stretch located between Porto Primavera Reservoir and Itaipu Reservoir (see Figure S1 in Supporting Information). This 230 km reach is accompanied by a wide floodplain (≤20 km) on the west margin and experiences a relatively predictable seasonal flood pulse influenced by several important free-flowing tributaries (e.g. Ivinheima and Baía Rivers) from December through April. The Upper Paraná floodplain is strongly affected by the consequences of basin-wide impoundments and other human activities, including alterations of inundation dynamics, community structure and ecosystem processes [22]–[25]. The functioning of the floodplain is critical for maintenance of regional biodiversity with approximately 4,500 species utilizing the floodplain [26].

**Figure 1:**
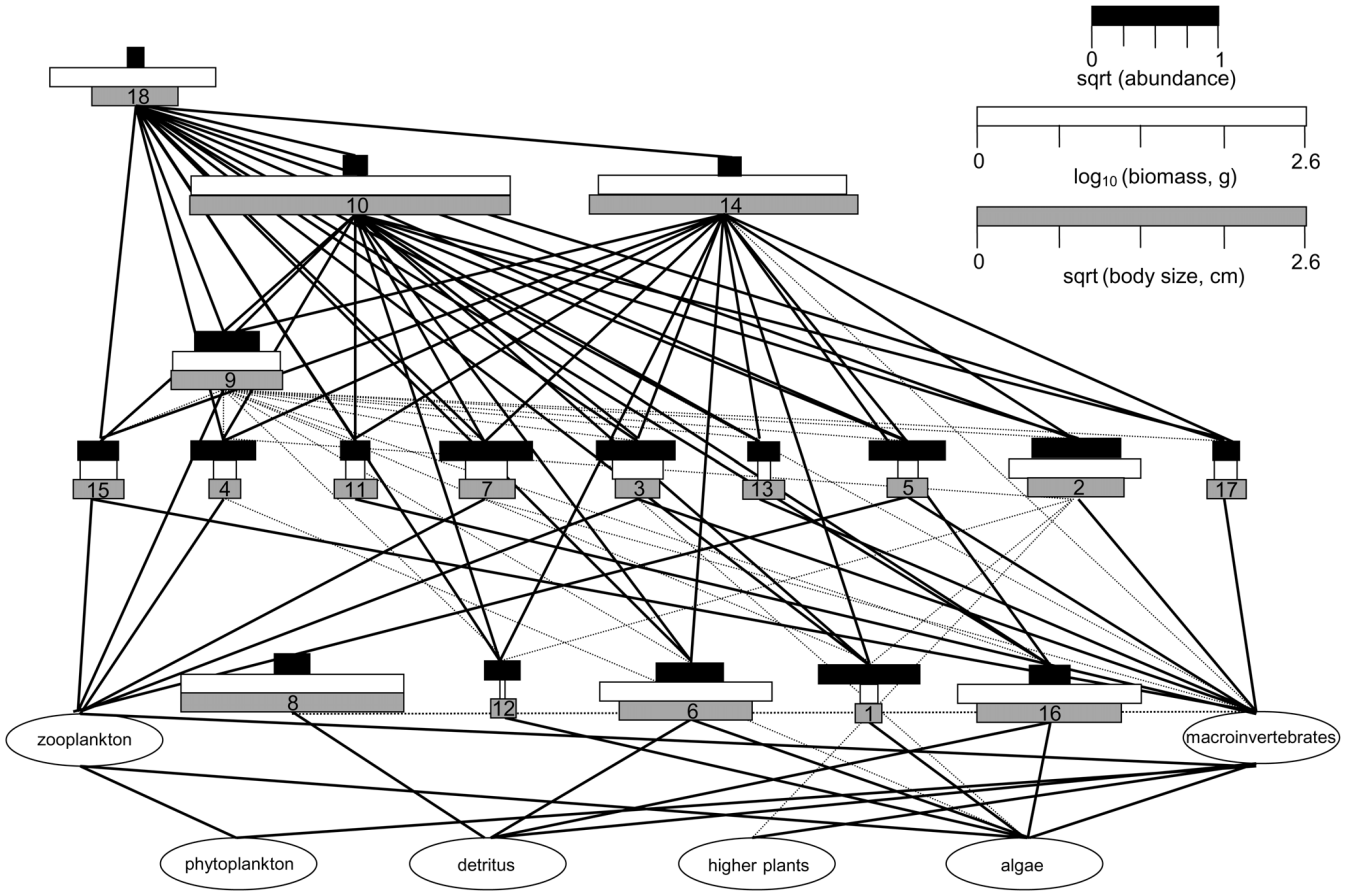
Expected food web for 18 fish species based on known trophic relationships (see Table S1). The width of black, white, and gray bars for each fish species (nodes) represent square root abundance, log_10_ mean biomass of individuals (g), and the square root mean body size (cm) of individuals from community sampling data, respectively. The number for each species is its ordered sum rank abundance (Appendix S1).

The LTER program has intensively studied biological, geophysical, and social aspects of the floodplain for more than a decade [23]. Fish assemblage data from floodplain lagoons collected as part of the LTER program were used to identify patterns of species relative abundances and provide the foundation for our experimental design. Fishes were collected by seine in the littoral zone (standardized by seine size, mesh, and sampling area) of isolated floodplain lagoons (n = 11) from 2000 to 2007. Isolated lagoons are distinguished from connected lagoons by the absence of a connection to the river during the low water period. Colonization occurs only during the high water period when the floodplain is inundated, and population dynamics are driven by factors occurring within each lagoon (i.e. no immigration or emigration) following isolation with declining water levels. Only samples from austral spring were considered so as to coincide with our study period and seasonality of lagoon isolation (i.e. low water season). Individual fishes (n = 7,447) representing 59 species were weighed and measured. Species richness of individual lagoons ranged from 3 to 19 ( = 10). Biodiversity experiments can enhance realism by using the full biodiversity gradient for a given ecosystem [27]. Therefore, we identified 2 species and 18 species as our low- and high-diversity endpoints with the midpoint of 10 species so as to match the range and average species richness observed for the lagoon fish species richness gradient. Species included in this design account for 82% of the total abundance of all fishes sampled and 47% of the total biomass of species with a mean standard length (SL) <20 cm (see Appendix S1). Although a few larger species occur in low abundance and contribute to large portions of residual biomass, seasonal flooding and isolation limit occurrence of these species to sporadic encounters in few lagoons. The 18 species included in the experiment represent the majority of ecomorphological diversity observed in floodplain fish assemblages, including seven trophic guilds (see Table S1) with various methods of resource capture and diet within and among guilds. Together, the 18 fish species and their prey resources comprise a complex food web with a high expected degree of connectivity (Figure 1).

**Figure 2:**
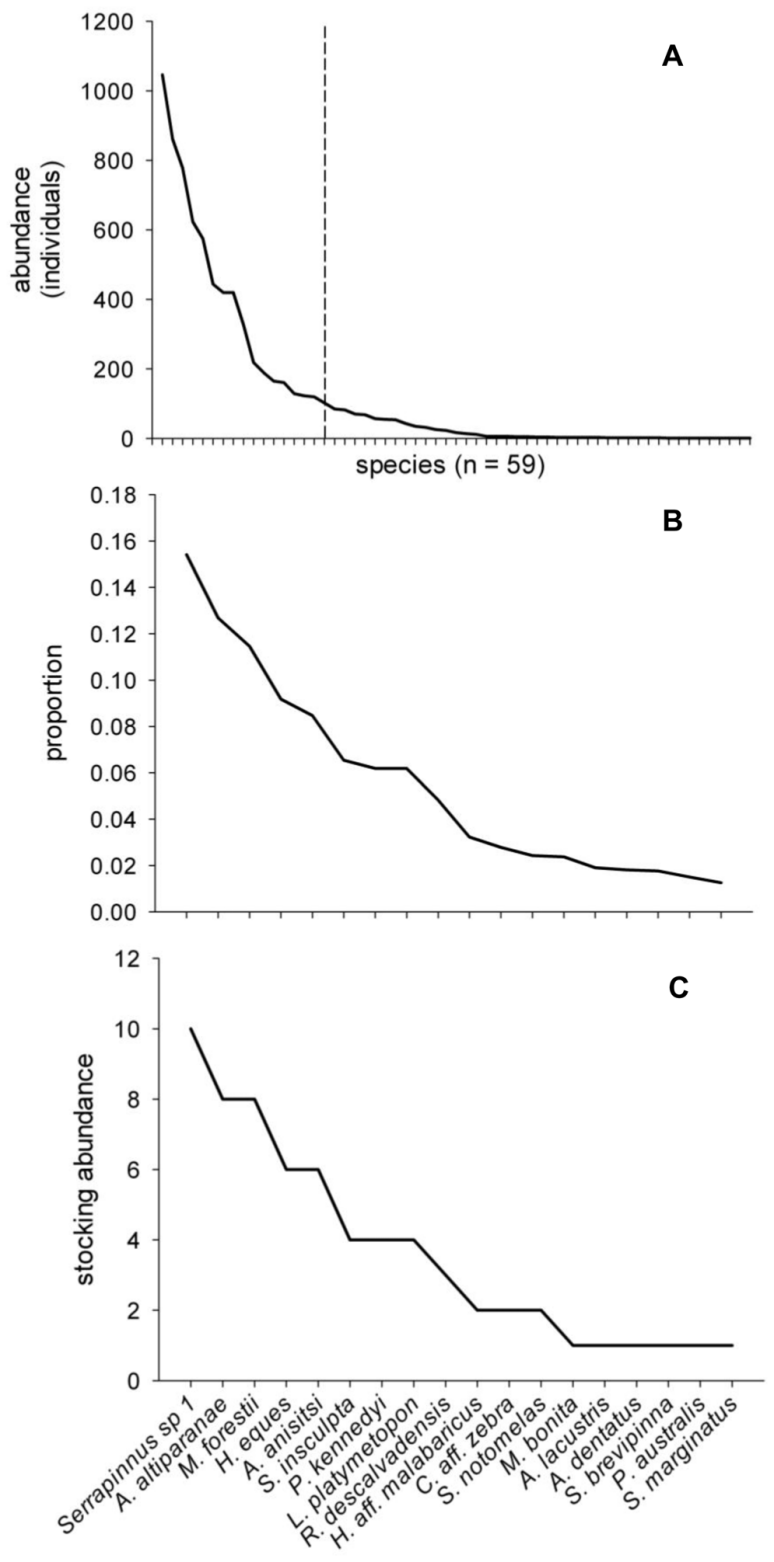
Rank abundance curve and example of stocking abundances. A) Rank abundance curve of 59 fish species derived from community sampling data from isolated lagoons (n = 11) in the Upper Paraná River floodplain during austral spring from 2000–2007. Species to the left of the dashed line represent the pool of species (n = 18) used in the experiment. B) Proportional rank abundance curve of the 18 species from (A) that provides the basis for experiment stocking abundances. C) Example of stocking abundances for the 18 species richness treatment (total 65 individuals per mesocosm).

To identify patterns of relative abundance, species were ranked and ordered according to their observed abundances by lagoon for each year. Species presence varied temporally among lagoons in this dynamic system, and species not present in a particular lagoon were assigned the median rank between the number of species present in that lagoon and the total number of species observed during the collection period (n = 59). This prevented a highly abundant species in a single lagoon from inflating its abundance across all lagoons over time by accounting for lagoons and years in which it was absent. Each species rank was then summed across lagoons over time to identify species that consistently occurred in most or all lagoons and with high relative abundances. Next, a rank abundance curve was generated based on the observed abundances of all species across all lagoons and time (Figure 2 A). Based on this curve, sequentially nested subsets of the high-diversity endpoint (18 species; Figure 2 B) were used as experimental assemblages representing sequential loss of rare species, with the middle experimental richness level equal to the mean species richness observed in the field data (10 species; Figure 2 C; see Experimental Design below and also [10] for a comparable approach).

Experimental stocking densities were informed by fish densities from littoral zones of isolated lagoons prior to the start of the experiment. Samples were collected for the same set of lagoons and using the same standardized methodology as described above, with the exception that one of the lagoons had gone dry and was therefore not sampled. Densities were highly variable, and ranged from <1 to more than 200 individuals per square meter. Fish densities are not only variable among lagoons but also over time, and are influenced by colonization history, time since isolation, and physicochemical characteristics [23]. Specifically, densities typically increase throughout the isolation period until extreme conditions and possibly complete desiccation result in high mortality. Stocking densities were set at 65 individuals based on surface area and volume of our mesocosms (see *Experimental venue*), which is well within the bounds observed in natural lagoons, and is close to long-term averages for isolated lagoons during austral spring under normal conditions (i.e. excluding extremely wet or dry years).

### Experimental Venue

Twenty-four 1000 L cylindrical polyethylene mesocosms (1.0 m high, 1.4 m in diameter) located at the Upper Paraná River Floodplain LTER field station were used as experimental units representing littoral habitats of isolated lagoons. Individual mesocosms were placed in a 6×4 design and randomly assigned to treatments (i.e. experimental assemblages). Mesocosms were discrete units but were otherwise open to climatic conditions. Mesocosms were filled with river water and sandy substrate from the Upper Paraná River adjacent to the floodplain. This stretch of the Upper Paraná River is oligotrophic from reduced sediment and nutrient transport caused by upstream impoundments, in contrast with isolated floodplain lagoons that generally have higher nutrient, phytoplankton, and zooplankton concentrations during the low water period due to local processes (e.g. decomposition of aquatic and riparian vegetation, resuspension of sediment by wind; [23]). To better simulate conditions found in isolated lagoons, 0.5 g of fertilizer and a concentrated slurry of phytoplankton (450 mL) and zooplankton (380 mL) taken from natural lagoons were added to each mesocosm. Phytoplankton and zooplankton were collected by filtering water through 20 µm mesh, concentrated, and homogenized prior to addition to each mesocosm. Following these additions, average initial concentrations of variables of interest among mesocosms better resembled concentrations observed in natural isolated lagoons during austral spring though with far lower variability (values reflect the mean and standard deviations for concentrations in mesocosms at the start of the experiment and values obtained from natural lagoons, respectively): total phosphorus (TP) (28 µg/L ±7; 88 µg/L ±92), total nitrogen (TN) (694 µg/L ±99; 458 µg/L ±198), phytoplankton (measured as chlorophyll *a* concentration, 12 µg/L ±6; 15 µg/L ±22), and zooplankton (15,375 individuals/m^3^±7828; field data reported separately for major taxonomic groups, e.g. approximate mean copepod density of 5,000 individuals/m^3^ and cladoceran density of 7,000 individuals/m^3^) [23]. To provide structure, floating macrophytes (*Eichhornia crassipes* and *Pistia stratiotes*; dominant macrophytes throughout the Upper Paraná River basin) were rinsed to remove epifauna, standardized for size and added to each mesocosm. Two clay bricks with 6 hollow partitions were also added to each mesocosm. Although artificial, bricks provided standardized benthic structure similar to that found in natural lagoons (e.g. woody debris and stones). Shade cloth (50% light penetration according to manufacturer specifications) covered all mesocosms and represented riparian reduction of direct solar irradiance. Mesocosms were open for aerial colonization by macroinvertebrates; however we did not quantify macroinvertebrates in this study. Fishes were collected by seine, gillnet, and electrofishing, and were placed in holding tanks prior to stocking to ensure survival and acclimation to experimental conditions. Fishes were stocked in experimental mesocosms over a 48 hour period, and individuals that succumbed to handling stress were replaced for the first 10 days of the experiment. Subsequent mortality was attributed to interactions occurring within the experiment and not from handling during stocking.

### Experimental Design

Experimental assemblages were structured based on losses of rare species using species ordered summed rank abundances. Assemblages were defined based on a standardized sequence of species loss along the lagoon fish species richness gradient such that the high- and low-diversity end-points and mean observed species richness based on field surveys were the bounds and midpoint for the treatments (18, 14, 10, 6, and 2 species; Table S2). To isolate the effect of species losses, we controlled for abundance (65 individuals per mesocosm) and evenness among experimental assemblages by proportionally distributing individuals from those species that were excluded from the prior assemblage (i.e. the four least abundant species) to remaining species in the subsequent assemblage such that the slope of the rank-abundance curve (i.e. evenness) was maintained constant (Figure 2). For example, when determining abundances for the 6-species assemblage, the nine individuals (totaled from the four least abundant species in the 10-species assemblage) were proportionally distributed among the six species in this assemblage (see Table S2 for stocking densities of each treatment). Thus, our experimental assemblages maintain natural patterns of dominance and rarity observed in an average lagoon, while isolating the effect of sequential loss of the rarest species. Treatments for this experiment are thus the starting or stocking assemblages as defined above, and assemblages were not artificially maintained during the experiment by additional stocking of individuals lost due to species interactions (i.e. predation or competition). Additionally, a treatment without fishes was included as a control. Due to the high morphological diversity of species included, we were unable to maintain biomass constant at the same time as abundance and evenness. Therefore, we accounted for potential effects of biomass among treatments by including it as a covariate in analyses. The experiment ran for three weeks and each treatment was replicated four times.

### Community and Ecosystem Responses over Time

Nine components of ecosystem structure and function were measured as response variables: change in fish assemblage structure, nutrient concentrations (TP and TN), phytoplankton density, zooplankton density, whole ecosystem metabolism [gross primary production (GPP) and net primary production (NPP)], benthic organic matter (BOM), and macrophyte biomass. All response variables were measured weekly except for fish assemblage structure and macrophyte biomass which were measured at the end of the experiment. Day 0 samples were collected after all fishes were stocked, and all subsequent weekly samples were collected on 7 day intervals based on that date. Water samples (500 mL) were taken from the center of each mesocosm (0.5 m depth) and analyzed for TP and TN using standard techniques [28]. Additional water samples (250 mL each) were taken from each mesocosm at five evenly distributed locations 30 cm below the water’s surface and pooled to form a 1.25 L sample from which phytoplankton (measured as chlorophyll *a*) and zooplankton were quantified. Each pooled sample was homogenized and 250 mL was filtered onto a 47 µm glass fiber filter for quantification of chlorophyll *a* concentration using standard spectrophotometric techniques. Remaining water (1 L) was filtered (64 µm sieve) and a 10% subsample was taken to estimate zooplankton density (number of individuals per m^3^). GPP and NPP were calculated from diel oxygen curves measured in the same location of each mesocosm over a 24 h period using a handheld YSI 85 dissolved oxygen probe. BOM was estimated as organic content ash free dry mass (AFDM) from a sediment core sampled from an *a priori* determined quadrat within each mesocosm using a plastic Petri dish (diameter 8.5 cm). Macrophyte biomass was estimated as the dry weight for each species at the end of the experiment.

An index of ecosystem multifunctionality was calculated based on average Z-scores across the seven response variables that were measured on a weekly basis [29]. To account for changes among response variables through time, Z-scores were calculated for each time step in each mesocosm to allow for all response variables to be measured on a common scale of standard deviation units for a given time step. The seven ecosystem functions measured in this experiment relate to the maintenance of primary production, secondary production, and nutrient cycling, therefore we assume higher values for these functions equates to higher overall ecosystem multifunctionality.

### Statistical Analyses

Fish assemblages were subject to multitrophic interactions, therefore we compared final fish assemblage structure in each mesocosm at the end of the experiment using non-metric multidimensional scaling (NMDS) in R (version 2.11.1). The analysis was conducted using Bray-Curtis similarities calculated from the species relative abundance by replicate matrix. Treatment effects on macrophyte biomass were compared using one-way ANCOVA with fish biomass initially included as a covariate. However, fish biomass was a nonsignificant covariate (*F*_1,17_ = 0.150, p = 0.703), therefore the analysis was performed using one-way ANOVA. The seven weekly-measured response variables were analyzed using mixed model, repeated measures ANOVA (rmANOVA). Mesocosms were treated as a random effect, treatment was the fixed effect, and fish biomass was included as a time-varying covariate (see below). An unstructured covariate matrix was used based on the lowest Akaike’s Information Criterion (AIC) value when testing against other covariate matrices. A Shapiro-Wilk (α = 0.05) test confirmed normality of residuals to meet the assumptions of ANOVA [30]. When normality was violated, response variables were log_10_ transformed to approach normality (NPP and BOM not transformed). Several response variables violated normality of residuals when viewed over the entire study period, due to varying responses of treatments over time. Therefore, normality of residuals was confirmed at each time step except for NPP and BOM on day 0 (p = 0.014 for both), phytoplankton on day 14 (p = 0.038), and nitrogen on day 21 (p = 0.008). We further investigated significant time interactions (i.e. simple main effects) using the SLICE command in SAS to test for treatment effects at each time step. All analyses were conducted in SAS 9.3 and SPSS 20.0.

Fish biomass was included as a time-varying covariate to test for effects of species richness after controlling for differences in biomass. Initial and final fish biomass were estimated based on species length-weight regressions calculated using the above-mentioned LTER data for fishes collected in isolated lagoons, and supplemented with measurements of individuals collected as part of this experiment. Fish biomass during the experiment was estimated using an exponential decay function (*y* = *ae^−bx^*) as resources were expected to decrease exponentially, rather than in a linear fashion throughout the experiment. This assumption is supported by trends among response variables indicating resource competition among lower trophic levels at the beginning of the experiment, and because predation of lower trophic levels was assumed to be maximized at the start of the experiment based on prey density. To aid in visual representation of response variables among treatments and over time after controlling for differences in fish biomass, response variables were regressed against fish biomass and residuals were saved and plotted.

### Direct Trophic Interactions among Response Variables

Strong direct trophic interactions exist among many of the response variables measured in this study. To investigate possible mechanisms driving observed variation in response variables over time, a second series of covariate analyses representing direct trophic interactions was explored using every possible covariate combination and analyzed using mixed model, rmANOVA. A backward selection approach was used to exclude models with nonsignificant covariates. Best-fitting models were then selected and interpreted based on the lowest Δ*_i_* Akaike’s Information Criterion (Δ*_i_* AIC = AIC*_i_* – AIC*_min_*; level of empirical support Δ*_i_* = 0–2 substantial, 4–7 considerably less, >10 essentially none) [31]. In order to use macrophyte biomass as a time varying covariate, values were estimated in a manner similar to fish biomass using initial and final dry mass, except that a linear model was used to estimate growth over time [32]. Initial macrophyte biomass was estimated as the combined average dry weight for each species, similar in size to those stocked in experimental mesocosms. As GPP and NPP were composite measures of whole ecosystem processes, direct trophic interaction covariate models were not explored.

## Results

### Community and Ecosystem Responses over Time

Fish assemblages changed over time, and mortality tended to increase with species richness, which corresponded with an increase in the number of piscivores present [Figure S2 and Table S3; ANOVA p = 0.111 for the full dataset and p = 0.016 when excluding outliers (one replicate each from 2, 6, and 10 species assemblages)]. However, a decrease in mortality was observed for the 18 species treatment even though this experimental assemblage had the highest number of piscivorous species. In this assemblage, intraguild predation by the piranha *Serrasalmus marginatus* on the smallscale pike characid *Acestrorhynchus lacustris* resulted in a decrease in the overall predation rate (the piranha consumed the caudal fin of this piscivorous species, resulting in mortality in three of the four replicates). For the single replicate of the 18 species treatment that lacked intraguild predation, overall mortality was comparable to that observed in the 14 species treatment (Table S3). Some mortality was also observed in treatments that lacked piscivores (i.e. 2 and 6 species assemblages). Ordination of the final species relative abundance by replicate matrix using NMDS indicated mortality affected the relative similarity in assemblage structure among treatments, but that among treatment variation generally exceeded within treatment variation (Figure S2).

**Figure 3:**
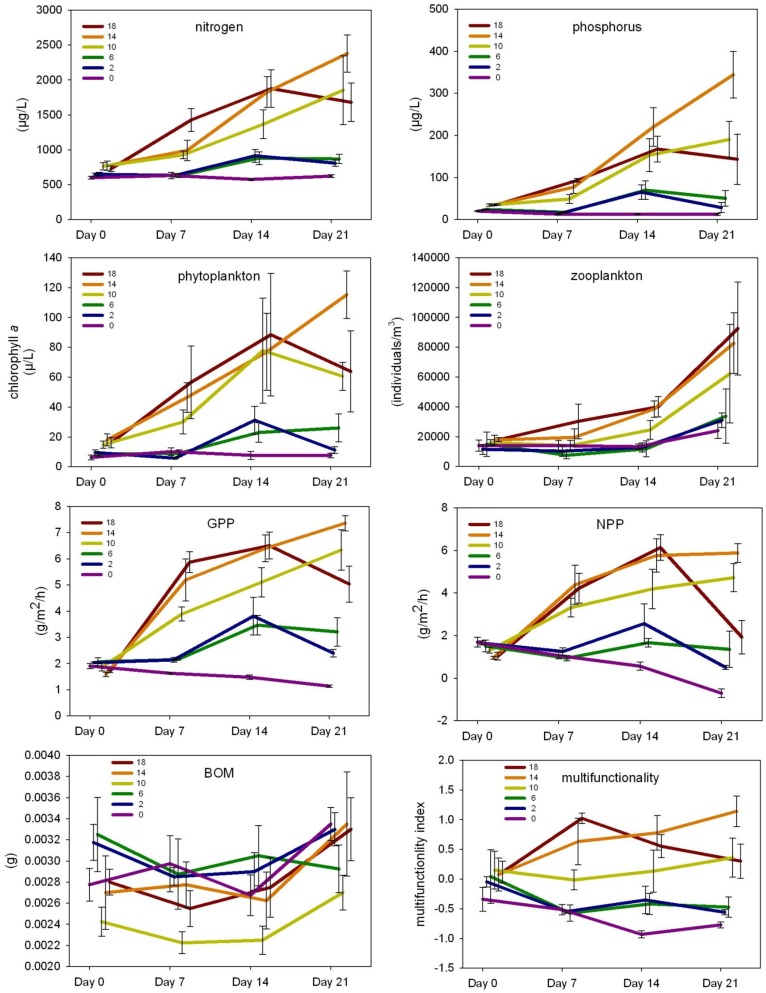
Mean and standard error of response variables (raw values) by treatment and over time.

Nutrient concentrations, phytoplankton density, zooplankton density, and whole ecosystem metabolism generally increased over time, and typically had higher values in treatments with more diverse experimental assemblages (10, 14 and 18 species) when compared to treatments with less diverse assemblages (0, 2 and 6 species; Figure 3). Ecosystem multifunctionality also tended to have higher values in more diverse treatments but did not necessarily increase over time; multifunctionality for 2 and 6 species assemblages remained relatively constant, 10 and 14 species assemblages increased over time, while the 18 species assemblage decreased at the end of the experiment. The multifunctionality index is consistent with combined trends observed for all other response variables (e.g. nutrients, phytoplankton, and whole ecosystem metabolism).

**Figure 4:**
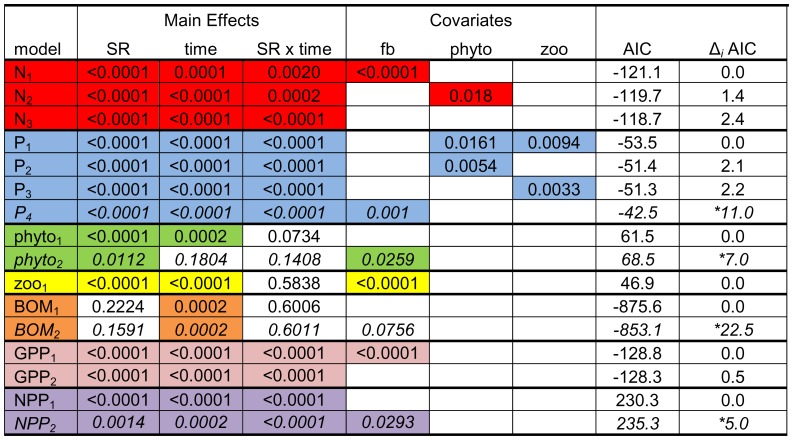
Best fitting models for each response variable calculated using mixed model, repeated measures ANOVA. Models investigated direct trophic interactions using every possible covariate combination as well as the effect of fish biomass (treatment covariate). Each row represents an individual model with response variables grouped by color (red = nitrogen, blue = phosphorus, green = phytoplankton, yellow = zooplankton, orange = benthic organic matter, pink = GPP, purple = NPP). For a given response variable, models are ranked by goodness of fit according to Δ*_i_* AIC value. *P*-values for the main effects (SR = species richness) and covariate(s) (fb = total fish biomass, phyto = phytoplankton, zoo = zooplankton) from ANOVA models are given within cells and shaded cells represent significant results (α = 0.05; full ANOVA results given in Table S3). Only models with Δ*_i_* AIC <3 are shown, except that models where fish biomass exceeded the <3 Δ*_i_* AIC threshold are still presented and denoted in italics and * next to Δ*_i_* AIC value. As GPP and NPP are composite measures of whole ecosystem processes, direct trophic interaction covariate models were not explored.

**Figure 5:**
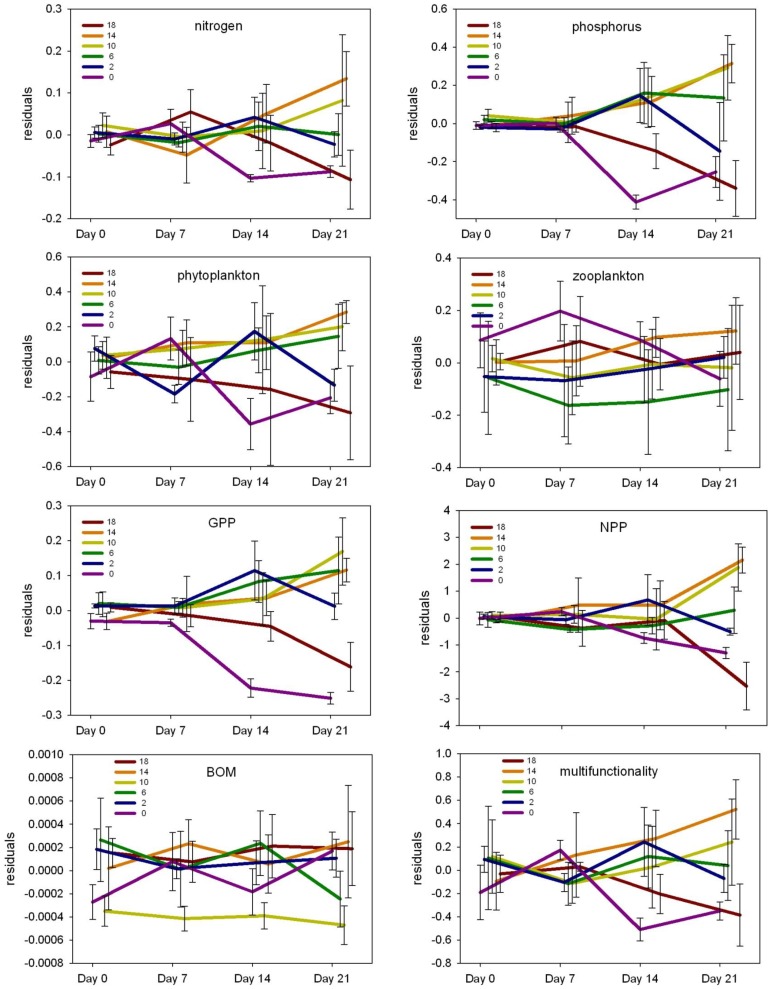
Mean and standard error of unstandardized residuals of response variables by treatment and over time. Residuals are from simple linear regression of the response variable and fish biomass, and therefore are measures of the response variable among treatments that control for the potentially confounding effect of fish biomass.

After controlling for the effects of fish biomass, significant species richness effects were observed for all response variables, with the exception of BOM (Figure 4; full ANOVA results are provided in Table S4). However, general increases over time among ecosystem responses were less evident among treatments after controlling for fish biomass (Figure 5). Most response variables for the control and 18 species assemblages generally decreased during the experiment, although increases in nutrients and phytoplankton were observed at the end of the experiment for the control. The 10 and 14 species assemblages, and 6 species assemblage to a lesser degree, exhibited increasing values for nutrient concentrations, phytoplankton, whole ecosystem metabolism and multifunctionality over time.

**Figure 6:**
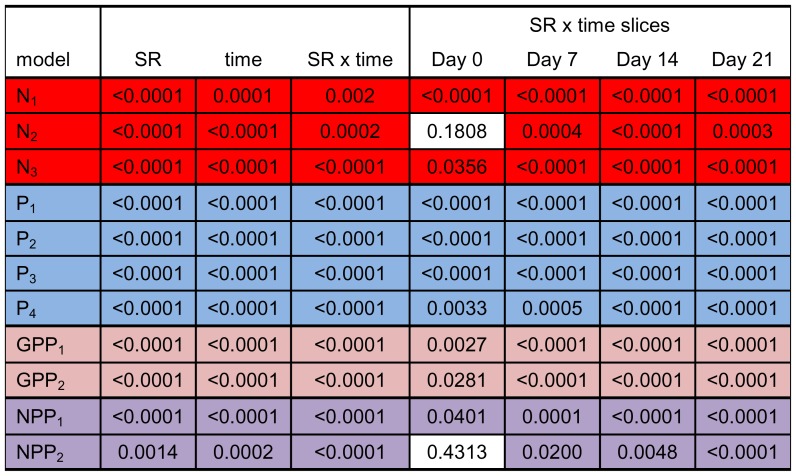
Slice effects for best fitting models for each response variable. Each row represents an individual model when a significant species richness (SR) by time interaction was observed with response variables grouped by color (red = nitrogen, blue = phosphorus, pink = GPP, purple = NPP). *P*-values for the main effects and time steps from ANOVA models are given within cells and shaded cells represent significant results (α = 0.05; full ANOVA results are provided in Table S3).

After controlling for fish biomass, nitrogen and phosphorus concentrations were significantly different among treatments and over time (p<0.0001; Figure 4), and had significant treatment by time interactions in which significant treatment effects were detected at all time steps (p<0.01; Figure 6). Phytoplankton and zooplankton densities differed among treatments (p = 0.011 and p<0.0001, respectively), and zooplankton differed over time (p<0.0001; Figure 4). After controlling for fish biomass, whole ecosystem metabolism (GPP and NPP) differed significantly among treatments (p<0.0001 and p = 0.0014, respectively) and over time (p<0.0001 and p = 0.0002, respectively; Figure 4). A significant treatment by time interaction was observed for GPP and NPP (both p<0.0001; Figure 4), where significant treatment effects (p<0.02; Figure 6) were detected at all time steps excluding the start of the experiment for NPP (i.e. day 0). No treatment effects were observed for BOM, and macrophyte biomass did not differ among treatments for either species (*E. crassipes: F*_5,18_ = 1.507, p = 0.237; *P. stratiotes*: *F*_5,18_ = 0.586, p = 0.710) or their combined weight (*F*_5,18_ = 1.504, p = 0.238).

### Effects of Direct Trophic Interactions on Response Variables

Total fish biomass alone best explained the differences in nitrogen concentration among treatments, with significant treatment and time effects and a treatment by time interaction (p<0.001, p = 0.0001, and p = 0.002, respectively; Figure 4), but phytoplankton density alone as covariate and the model with no covariates had similar AIC values with similar significant main effects (Figure 4). Significant treatment effects were observed at all time steps for these models (Figure 6). For phosphorus concentration, the best-fitting model included phytoplankton and zooplankton densities together as covariates (p = 0.016 and p = 0.009, respectively; Figure 4), with significant treatment and time effects and a treatment by time interaction (all p<0.001; Figure 4). Other suitable models included zooplankton density alone and phytoplankton density alone as covariates with similar main effects. Significant treatment effects were detected at all time steps for these phosphorus models (Figure 6). The best-fitting model for phytoplankton density contained no covariates and had significant treatment and time effects (p<0.0001 and p = 0.0002, respectively; Figure 4). For zooplankton density, fish biomass best explained differences among treatments (Figure 4), with significant treatment and time effects (both p<0.0001; Figure 4). The best fitting model for BOM did not include any covariates and had a significant time effect (p = 0.0002; Figure 4).

## Discussion

### Ecosystem Consequences of Species Loss

Nonrandom loss of rare fish species from our diverse experimental food webs affected various aspects of ecosystem structure and function. Differences in mortality among treatments resulted in convergence of fish assemblage structure among some treatments. In general, more specious assemblages (10, 14, and 18 fish species), and notably the only assemblages that included piscivores, were associated with higher nutrient concentrations, phytoplankton and zooplankton densities, and whole ecosystem metabolism when compared with less diverse assemblages (0, 2 and 6 fish species). It is also worth noting that some variation was observed among treatments at the start of the experiment for several response variables, which might indicate differing starting conditions among mesocosms. As mentioned above, fishes were stocked over a 48 hour period, prior to the “day 0” sampling event, therefore, variability in response variables among treatments at the start of the experiment likely represents the early onset of a treatment-dependent response (also supported by the generally lower significance compared with subsequent time steps for each response variable). After accounting for the potential influence of fish biomass, species richness significantly influenced all measured ecosystem responses with the exception of BOM and macrophyte biomass. Nutrient concentrations, phytoplankton, whole ecosystem metabolism, and multifunctionality still increased over time in the 10 and 14 species treatments, but notable decreases were present for most ecosystem responses in the 18 species treatment. Overall, losses of fish diversity affected ecosystem structure and function through various direct (e.g. predation) and indirect (e.g. nutrient-mediated effects) pathways, suggesting loss of rare species may have profound effects across multiple components of diverse multitrophic ecosystems. This finding is analogous to the “cornerstone” role of rare species in ecosystem functioning described in a previous study [17].

Effects of biodiversity on ecosystem functioning are often attributed to complementarity and the selection effect as primary mechanisms [2]. That is, increases in ecosystem functioning with increasing diversity are the result of limited functional redundancy among species or that higher diversity increases the probability of “selecting” taxa with greater individual influences on ecosystem functioning. This is especially the case for studies that manipulate diversity within a single trophic level, although these mechanisms have also been shown to operate in simplified multitrophic systems [33]. In ecosystems with natural patterns of commonness and rarity, the relative abundance or biomass of species may mediate the roles of complementarity and the selection effect (i.e. species composition) via enhancement or dilution of species’ per capita or per biomass contributions to overall ecosystem functioning [13]. Furthermore, the strength by which resource complementarity and the selection effect govern ecosystem functioning may be weakened in complex food webs by the numerous trophic interactions and indirect effects that are present [34]. For instance, Downing and Leibold [8] manipulated diversity across multiple trophic positions in experimental pond food webs and found that changes in species richness and composition affected ecosystem processes primarily in an indirect manner by altering the abundance and composition of non-manipulated components of the food web. The authors suggest that even small shifts in abundance or composition can translate into larger ecosystem effects through various trophic interactions and indirect effects present in more complex multitrophic systems. Indirect effects in multitrophic systems limit the ability to attribute ecosystem-level consequences to complementarity and the selection effect, which are more readily interpretable for biodiversity within a single trophic level and directly associated ecosystem responses. Similarly, in this experiment, we manipulated species richness within and among trophic levels, whereas other multitrophic BEF studies typically manipulate diversity within a single trophic level [20], [33], [35] but see [34], [36]. Therefore, as loss of the rarest species occurred simultaneously across multiple components of this complex food web, observed influences on ecosystem processes and multifunctionality are likely the combined result of indirect effects of manipulated taxa (i.e. loss of multiple rare fish species) on non-manipulated components (e.g. phytoplankton and zooplankton abundance and composition) in addition to direct effects (e.g. intraguild predation) attributable to the manipulated loss of species richness.

Species identity can strongly influence different components of ecosystem functioning such as nutrient cycling [11], [37], belowground community composition [38] and pest suppression [39], even in multitrophic systems [38]. However, because our aim was to explore realistic scenarios of biodiversity loss in a highly diverse multitrophic study system, we knowingly made a trade-off between a fully factorial design and high species richness that well-represented lagoon assemblages in our system. This trade-off means the role of individual species cannot be fully disentangled from diversity effects. That being said, complementary lines of evidence give us confidence in attributing some ecosystem effects to the piranha *S. marginatus*. The occurrence of intraguild predation by the piranha on the smallscale pike characid *A. lacustris* in the 18 species assemblages contributed to differences in mortality and nutrient concentrations among richness levels as well as significant treatment interactions over time. Predators can exert strong influences on nutrient cycling directly though excretion, egestion, and the translocation of nutrients and indirectly through non-lethal predator-prey interactions [40]. In this case, mortality and decomposition of the piscivorous smallscale pike characid at the beginning of the experiment in the 18 species treatment likely caused the early increase in nitrogen and phosphorus concentrations. Subsequent reduced predation over the remainder of the experiment (i.e. as compared with the 14 species treatment that lacked the piranha and was the only other treatment including the smallscale pike characid) appears to have resulted in a lower rate of nutrient remineralization from predator excretion. These nutrient-mediated effects may also explain subsequent decreases in phytoplankton densities, whole ecosystem metabolism, and multifunctionality through time in the 18 species treatment. Although our isolated experimental venue may be expected to result in a greater or more immediate impact of nutrient-mediated effects on ecosystem function, previous field studies on tropical fish assemblages have demonstrated significant and localized biogeochemical “hotspots” due to consumer-mediated nutrient dynamics [41], [42]. Thus, our results support the idea that species identity and intraguild predation in diverse predator assemblages can strongly influence the structure and function of an ecosystem through direct and indirect pathways [35]. It is worth noting that the piranha *S. marginatus* is not native to the Upper Paraná River floodplain but has greatly increased in abundance and displaced the less aggressive native piranha *Serrasalmus maculatus* [43], with unknown and potentially important community and ecosystem-level consequences.

Mortality was also observed in treatments that lacked piscivores (experimental assemblages with 2 and 6 fish species). In these treatments, mortality may be attributed to competition for limiting resources (i.e. phytoplankton, zooplankton) early in the experiment. Although our experimental design does not allow for direct evaluation of competitive interactions, we noted some individuals of algivorous and zooplanktivorous species appeared emaciated early in the experiment when fish densities were highest (i.e. before mortality) and algae and zooplankton densities were lowest. Similar transient patterns may be expected in natural lagoons directly following isolation from the main channel (analogous to the start of the experiment) since primary and secondary production has been shown to be positively related to water residence time (time since isolation from the main channel) [44], [45]. Therefore, in these closed systems (natural isolated lagoons and mesocosms) a lag period in primary and secondary production may be expected before sustainable populations are reached. Alternatively, non-predation mortality may also be a result of the unnatural conditions in the mesocosms and handling stress during stocking, though we expect such effects to be very minimal because the mesocosms contained water, substrates and plants from the natural environment, we replaced dead individuals at the beginning of the experiment, and fishes were observed performing foraging, schooling and habitat-associated behaviors as expected based on knowledge of these species in the natural environment.

In addition to the likely influence of intraguild predation on nutrient cycling, loss of rare species affected phosphorus concentrations even after accounting for fish biomass and phytoplankton and zooplankton densities (significant covariates in the direct trophic interactions models). Loss of rare species also significantly affected both phytoplankton and zooplankton densities, which means that species richness exhibited both direct and indirect effects on phosphorous concentrations. Direct effects of fishes on phosphorus cycling have been described in other Neotropical ecosystems. A small characid (*Astyanax aeneus*) was found to be a keystone phosphorus recycler in nutrient-poor streams by mediating phosphorus input from terrestrial subsidies [46]. Alternatively, armored catfish (Loricariidae) can influence nutrient cycling via sequestration of phosphorus required for maintenance of their armor [37]. As our experimental assemblages contained species closely related to those mentioned above (i.e. *Astyanax altiparanae* and *Loricariichthys platymetopon*), possible countervailing interactions may have arisen between the provisioning of phosphorus by *A. altiparanae* and sequestration of phosphorus by *L. playtmetopon*. Although difficult to discern, it is likely that *L. playtmetopon* had greater influences on phosphorus concentrations as terrestrial subsides available to *A. altiparanae* in our experimental venue are expected to be less than those observed in natural systems with greater riparian vegetation. We further speculate that decreasing trends in phosphorus observed in the 18 species treatment after accounting for differences in fish biomass may also in part be due to phosphorus sequestration by *L. playtmetopon* as this treatment contained the highest number of individuals.

Despite these plausible examples of the disproportionate influence of a species on a single or a few ecosystem processes, the multifunctionality of an ecosystem likely depends on multiple species contributing to different functions [18], [20]. Tradeoffs among functions can exist as more individual functions or more diverse functions are considered [20]. In our experiment, response variables represent nutrient cycling (N and P concentrations), primary production (phytoplankton density), secondary production (zooplankton density), organic matter accumulation (BOM) and ecosystem metabolism (NPP and GPP). Similar to other studies [18], [20], [29], multifunctionality increased with species richness (or from our context, decreased with loss of rare species), with the exception of the 18 species treatment later in the experiment. We interpret this pattern as reflecting a positive correlation between our response variables due to a dominant role of bottom-up effects. The primary exception to this pattern is the 18 species treatment where intraguild predation resulted in lower nutrient remineralization later in the experiment.

Although we interpret strong ecosystem effects of predators via nutrient remineralization, the loss of species across different positions of the food web may explain why certain trophic guilds did not influence ecosystem responses in a strong top-down manner. For example, trophic cascades have been observed in several studies manipulating species richness [35], [47], but our analyses revealed differences in phytoplankton and zooplankton densities among treatments were not related to algivore or zooplanktivore biomass. Since entire trophic guilds were not lost until low levels of species richness (i.e. guilds were represented by at least one species across most treatments), remaining species within these trophic guilds may have dampened the effects of species loss on overall ecosystem function through compensatory dynamics and food web complexity [48], [49].

Previous studies incorporating nonrandom loss of rare species yielded mixed results due in part to the relative contribution of rare species to the ecosystem process measured [10], [11], [13], [16], [17]. The relative contribution of a species to ecosystem functioning may diminish as multiple ecosystem functions are considered and/or are integrated into a single index of multifunctionality. Additionally, the influence of any one species in a multitrophic system will depend on the strength of interactions with other species and the cumulative direct and indirect pathways by which it may influence a given ecosystem process. In our experiment, loss of rare species significantly affected multiple aspects of ecosystem functioning, demonstrating that decreases in fish diversity, even by loss of comparatively minor components of an assemblage, can collectively influence the overall structure and functioning of multitrophic ecosystems.

### Caveats and Considerations

A few caveats need to be revisited when interpreting the results of this experiment. Foremost, and as indicated above, are the trade-offs associated with experimental designs that affect the ability to fully disentangle the effects of species identity and/or functional guilds from effects of species richness. A fully-factorial design that retained our realistic high-diversity end-point would require 262,143 treatments; clearly, such an experiment is not feasible, even for smaller-bodied non-vertebrate taxa. For this reason, experiments that include high species or functional group richness often employ designs that randomly select species from a species pool to populate treatments (e.g. richness levels) while controlling for the effect of species identity [8], [50], [51]. While this allows for a controlled test of species richness effects, it precludes such realistic scenarios of biodiversity loss that was the primary objective of this study.

Other experimental designs may retain realism in high diversity or multitrophic ecosystems by directly removing species or functional groups of interest according to a relevant scenario (e.g. loss rare species, as in [17] for a rocky intertidal ecosystem). In both [17] and our study, additional lines of evidence allowed for speculation regarding the key roles played by certain species and interactions couched within the role of diversity loss in general (e.g. the piranha and intraguild predation in our study and the seaweed *Ascophyllum* and facilitation in [17]). While such studies are not fully factorial and cannot unambiguously isolate species identity vs. species richness effects, previous research has shown that ecosystem consequences of biodiversity can be substantially different whether evaluated using randomized or ordered experimental assemblages [10], [12]. For example, similar to our study, [10] used a nested subsets design informed by natural assemblages and found invasion resistance of experimental grasslands to be significantly affected by species loss, in contrast with results from a randomized design employed in previous studies for this same system. Although randomized designs may isolate the species richness effect, they may lack relevance to natural systems, often with significant consequences for interpretation [10], [12]. Our study thus favored a realistic assemblage design, along with a measured attempt to isolate key mechanisms via statistical approaches and complementary data.

The integration of multitrophic perspectives and realistic diversity scenarios in BEF research is yielding new insights into the generality of findings from foundational BEF research. However, increasing the complexity of interactions and the number of species strains the ability of researchers to conduct fully-factorial experiments, especially with relatively larger-bodied and longer-lived organisms (e.g. vertebrates) in highly diverse regions such as the tropics. In addition to removal experiments and “in silico” simulations [27], we advocate a food web module approach [52] to understand the roles of key species and trophic guilds in addition to biodiversity per se on ecosystem functioning in a multitrophic context. For example, research to complement this study could focus on the role of native and non-native piranhas in mediating top-down and bottom-up effects via intraguild predation, or the influence of detritivore diversity on ecosystem function and food web stability [53] in relation to key traits that affect susceptibility to predation (e.g. body size, presence of armor) and energy flow and nutrient cycling (e.g. biomass, stoichiometry). As part of this integrated module approach, combined mesocosm and field experiments could further elucidate the mechanisms by which diversity effects manifest while also evaluating the role of experimental venue and spatial or temporal scales on observed relationships. Emerging trends from other BEF experiments suggest diversity effects may grow stronger with time [9], and interpretations from the present mesocosm study would be validated should field manipulations yield similar findings.

## Supporting Information

Figure S1 **Location of the Upper Paraná River and its floodplain, associated environmental protection areas, and extent of the LTER site.** The floodplain extends up to 20 km from the western margin of the Paraná River, primarily in the area of influence of the Ivinheima and Baía rivers.
(PDF)

Figure S2 **Non-metric multidimensional scaling of fish assemblages at the beginning and end of the experiment.** Initial assemblage structures for each treatment are indicated by open symbols, and the dot and dashed line denote the direction of change from initial to final assemblage structure for each treatment. Top left: mean and standard deviation of fish mortality at the end of the experiment for each treatment [excluding outliers (one replicate each from 2, 6, and 10 species assemblages); ANOVA p = 0.016].
(PDF)

Table S1 **Diet composition and trophic guild classification of 18 species used in experimental manipulations based on primary literature†.** Black and grey fill represent primary and secondary diet item, respectively.
(PDF)

Table S2 **Species composition, trophic guild assignment, and initial biomass comprising each experimental diversity treatment.** Values for each species at each diversity level are stocking abundances in experimental mesocosms. In the highest diversity treatment (n = 18), the species rank abundance curve is proportional to field data from isolated lagoons scaled down to 65 individuals (see Figure 2 C). For subsequent treatments, species are excluded based on their summed rank abundance, fitted to the original abundance curve (holding 65 individuals constant) by proportional addition of individuals to remaining species to maintain the natural pattern of dominance and rarity. Trophic guild assignment is based on previous research (see Table S1): (A) algivore, (D) detrivore, (D–A) detrivore/algivore, (I) insectivore, (P) piscivore, (Z) zooplanktivore, (Z–I) zooplanktivore-insectivore.
(PDF)

Table S3 **Initial and final abundances and biomasses for fish species in each experimental replicate.** Biomass values (g) are in parentheses.
(XLSX)

Table S4 **Mixed model, repeated measures ANOVA for each response variable and associated covariate models with direct trophic interactions.** When a significant treatment by time interaction was observed, SLICE effects are reported. Significant results in bold (α = 0.05).
(PDF)

Appendix S1 **List of species present from community sampling data from isolated lagoons in the Upper Paraná River floodplain (n = 11) during 2000–2007 austral springs.** Species above the dashed line represent the pool of species (n = 18) used in experimental treatments. Eighteen species represent 82% of the total abundance and 47% of total biomass of individuals with mean SL <20 cm.
(PDF)
