# Supplementary material for: Loss of Rare Fish Species from Tropical Floodplain Food Webs Affects Community Structure and Ecosystem Multifunctionality in a Mesocosm Experiment
Source: PLoS One. 2014 Jan 8;9(1):e84568. doi: 10.1371/journal.pone.0084568 (PMC3885587; doi:10.1371/journal.pone.0084568)
Supplement: Figure S1 — Location of the Upper Paraná River and its floodplain, associated environmental protection areas, and extent of the LTER site. The floodplain extends up to 20 km from the western margin of the Paraná River, primarily in the area of influence of the Ivinheima and Baía rivers. (PDF) [file pone.0084568.s001.pdf]

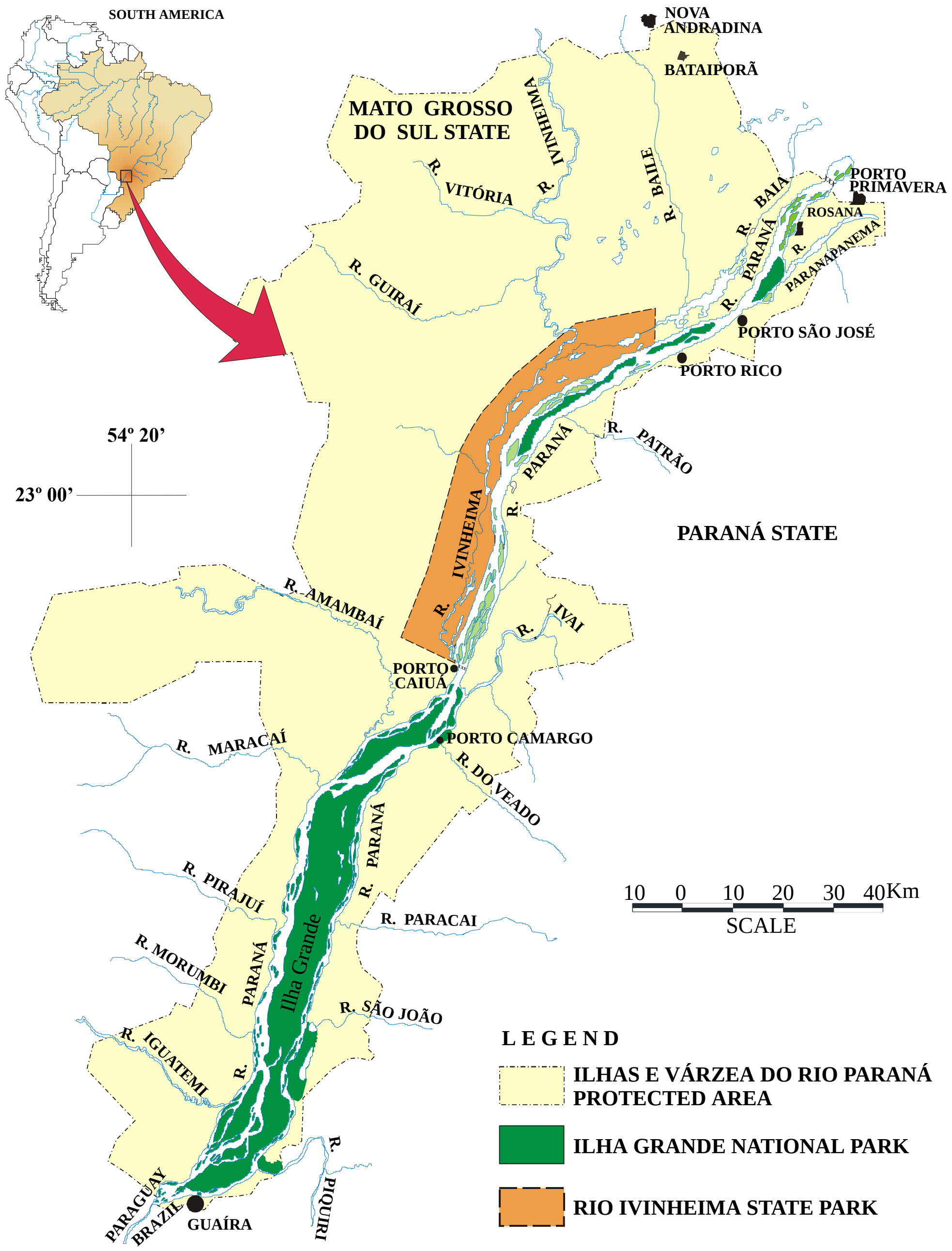

Figure S1. Location of the Upper Paraná River and its floodplain, associated environmental protection areas, and extent of the LTER site. The floodplain extends up to 20 km from the western margin of the Paraná River, primarily in the area of influence of the Ivinheima and Baía rivers.
