# Supplementary material for: Loss of Rare Fish Species from Tropical Floodplain Food Webs Affects Community Structure and Ecosystem Multifunctionality in a Mesocosm Experiment
Source: PLoS One. 2014 Jan 8;9(1):e84568. doi: 10.1371/journal.pone.0084568 (PMC3885587; doi:10.1371/journal.pone.0084568)
Supplement: Figure S2 — Non-metric multidimensional scaling of fish assemblages at the beginning and end of the experiment. Initial assemblage structures for each treatment are indicated by open symbols, and the dot and dashed line denote the direction of change from initial to final assemblage structure for each treatment. Top left: mean and standard deviation of fish mortality at the end of the experiment for each treatment [excluding outliers (one replicate each from 2, 6, and 10 species assemblages); ANOVA p = 0.016]. (PDF) [file pone.0084568.s002.pdf]

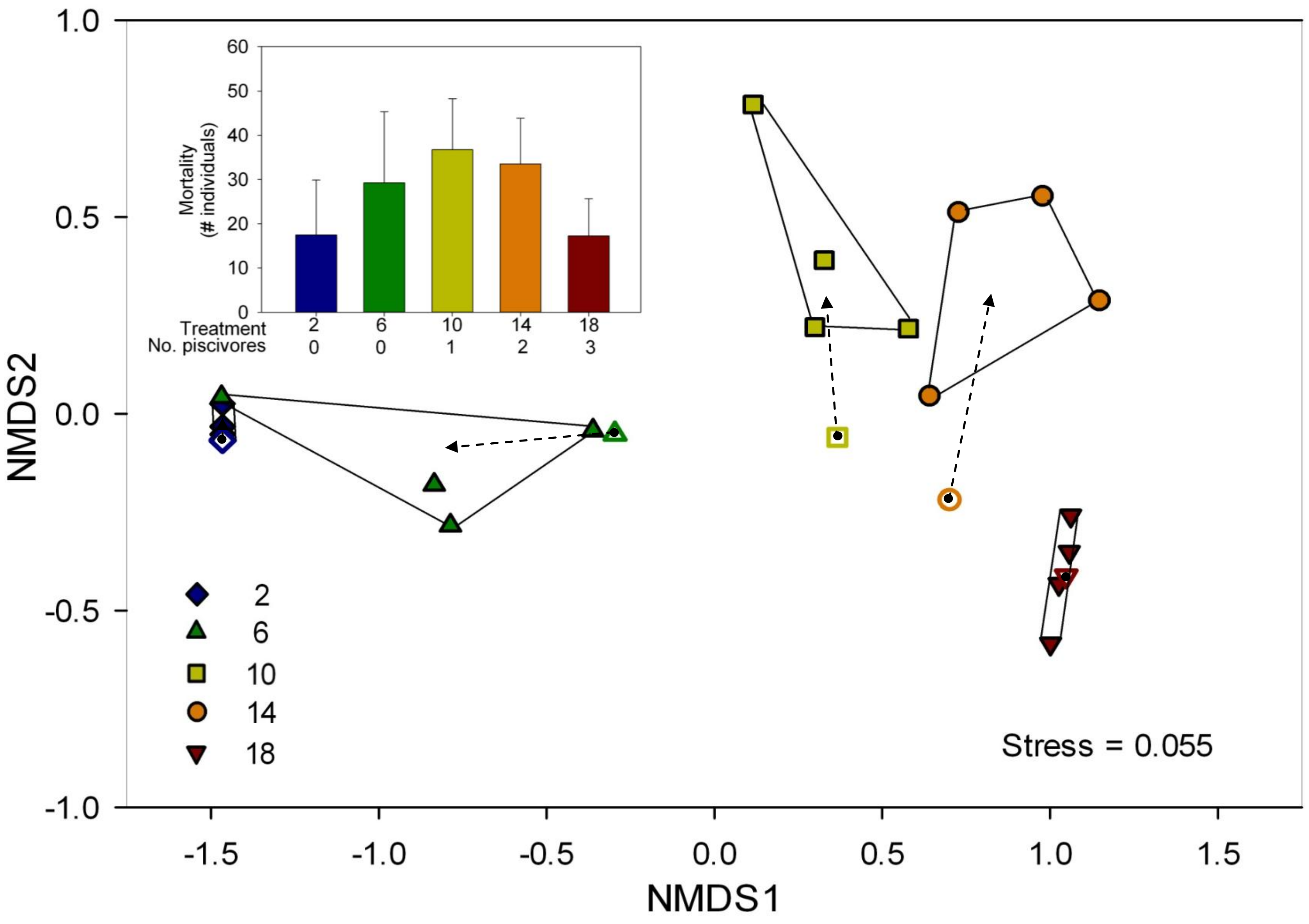

Figure S2. Non-metric multidimensional scaling of fish assemblages at the beginning and end of the experiment. Initial assemblage structures for each treatment are indicated by open symbols, and the dot and dashed line denote the direction of change from initial to final assemblage structure for each treatment. Top left: mean and standard deviation of fish mortality at the end of the experiment for each treatment [excluding outliers (one replicate each from 2, 6, and 10 species assemblages); ANOVA p = 0.016].
