# Supplementary material for: Loss of Rare Fish Species from Tropical Floodplain Food Webs Affects Community Structure and Ecosystem Multifunctionality in a Mesocosm Experiment
Source: PLoS One. 2014 Jan 8;9(1):e84568. doi: 10.1371/journal.pone.0084568 (PMC3885587; doi:10.1371/journal.pone.0084568)
Supplement: Table S1 — Diet composition and trophic guild classification of 18 species used in experimental manipulations based on primary literature†. Black and grey fill represent primary and secondary diet item, respectively. (PDF) [file pone.0084568.s003.pdf]

Table S1. Diet composition and trophic guild classification of 18 species used in experimental manipulations based on primary literature†. Black and grey fill represent primary and secondary diet item, respectively.

Species	Trophic guild	Zoo-plankton	Aquatic/ terrestrial insects	Fishes	Algae	Higher plants	Detritus/ sediment
Serrapinnus sp 1*	algivore				■		
Astyanax altiparanae	insectivore		■	▒		▒	
Moenkhausia forestii	zooplanktivore/insectivore	■	■		▒		
Hyphessobrycon eques	zooplanktivore	■			▒		
Aphyocharax anisitsi	zooplanktivore/insectivore	■	■				
Steindachnerina insculpta	detrivore/algivore				■		■
Psellogrammus kennedyi	zooplanktivore/insectivore	■	▒				
Loricariichthys platymetopon	detrivore		▒				■
Roeboides descalvadensis	zooplanktivore	■	▒	▒			
Hoplias aff. *malabaricus*	piscivore			■			
*Characidium*aff. *zebra*	insectivore		■				
Serrapinnus notomelas	algivore				■		
Moenkhausia bonita	insectivore		■				
Acestrorhynchus lacustris	piscivore		▒	■			
Aphyocharax dentatus	zooplanktivore/insectivore	■	■				
*Steindachnerina brevipinna**	detrivore/algivore				■		■
Pyrrhulina australis	insectivore		■				
Serrasalmus marginatus	piscivore		▒	■			

†Diet data from [23,54-61]

* Diet and trophic guild classification based on congener

23. Thomaz SM, Agostinho AA, Hahn NS (2004) The upper Paraná River and its floodplain – physical aspects, ecology, and conservation. Leiden, The Netherlands: Backhuys Publishers. 393 p.

54. Benine RC, Castro RMC, Sabino J (2004) *Moenkhausia bonita*: a new small characin fish from the Rio Paraguay basin, southwestern Brazil (Characiformes: Characidae). Copeia 2004: 68-73.

55. Correâ CE, Hahn NS, Delariva RL (2009) Extreme trophic segregation between sympatric fish species: the case of small sized body *Aphyocharax* in the Brazilian Pantanal. Hydrobiologia 635: 57-65.

56. Crippa VEL, Hahn NS, Fugi R (2009) Food resource used by small-sized fish in macrophyte patches in ponds of the upper Paraná river floodplain. Acta Sci Biol Sci 31: 119-125.

57. Esteves KE, Lobo AVP, Faria MDR (2008) Trophic structure of a fish community along environmental gradients of a subtropical river (Paraitinga River, Upper Tietê River Basin, Brazil). Hydrobiologia 598: 373–387.

58. Graca WJ, Pavanelli CS (2007) Peixes da planície de inundação do alto rio Paraná e áreas adjacentes. Maringá, Brazil: Editora do Universidade Estadual de Maringá.

59. Pelicice FM, Agostinho AA (2006) Feeding ecology of fishes associated with *Egeria* spp. patches in a tropical reservoir, Brazil Ecol Freshw Fish 15: 10–19.

60. Peretti D, Andrian IF (2004) Trophic structure of fish assemblages in five permanent lagoons of the high Paraná River floodplain, Brazil. Environ Biol Fish 71: 95-103.

61. Russo MR, Hahn NS (2006) Importance of zooplankton in the diet of a small fish in lagoons of the upper Paraná River floodplain, Brazil. Acta Limnol Bras 18: 357-366.
