# Supplementary material for: Loss of Rare Fish Species from Tropical Floodplain Food Webs Affects Community Structure and Ecosystem Multifunctionality in a Mesocosm Experiment
Source: PLoS One. 2014 Jan 8;9(1):e84568. doi: 10.1371/journal.pone.0084568 (PMC3885587; doi:10.1371/journal.pone.0084568)
Supplement: Table S2 — Species composition, trophic guild assignment, and initial biomass comprising each experimental diversity treatment. Values for each species at each diversity level are stocking abundances in experimental mesocosms. In the highest diversity treatment (n = 18), the species rank abundance curve is proportional to field data from isolated lagoons scaled down to 65 individuals (see Figure 2 C). For subsequent treatments, species are excluded based on their summed rank abundance, fitted to the original abundance curve (holding 65 individuals constant) by proportional addition of individuals to remaining species to maintain the natural pattern of dominance and rarity. Trophic guild assignment is based on previous research (see Table S1): (A) algivore, (D) detrivore, (D–A) detrivore/algivore, (I) insectivore, (P) piscivore, (Z) zooplanktivore, (Z–I) zooplanktivore-insectivore. (PDF) [file pone.0084568.s004.pdf]

Table S2. Species composition, trophic guild assignment, and initial biomass comprising each experimental diversity treatment. Values for each species at each diversity level are stocking abundances in experimental mesocosms. In the highest diversity treatment (n =18), the species rank abundance curve is proportional to field data from isolated lagoons scaled down to 65 individuals (see Figure 2 C). For subsequent treatments, species are excluded based on their summed rank abundance, fitted to the original abundance curve (holding 65 individuals constant) by proportional addition of individuals to remaining species to maintain the natural pattern of dominance and rarity. Trophic guild assignment is based on previous research (see Table S1): (A) algivore, (D) detrivore, (D-A) detrivore/algivore, (I) insectivore, (P) piscivore, (Z) zooplanktivore, (Z-I) zooplanktivore-insectivore.

Species	Diversity Level						Trophic Guild	Rank Abundance
No. species	0	2	6	10	14	18		
No. trophic guilds	0	2	5	7	7	7		
Biomass (g)	0	91	115	442	565	632		
Serrapinnus sp. 1		45	27	18	13	10	A	1
Astyanax altiparanae		20	16	12	10	8	I	2
Moenkhausia forestii			10	10	9	8	Z-I	3
Hyphessobrycon eques			5	7	7	6	Z	4
Aphyocharax anisitsi			4	6	5	6	Z-I	5
Steindachnerina insculpta			3	3	5	4	D-A	6
Psellogrammus kennedyi				3	4	4	Z-I	7
Loricariichthys platymetopon				2	3	4	D	8
Roeboides descalvadensis				2	2	3	Z	9
Hoplias aff. *malabaricus*				2	2	2	P	10
Characidium aff. *zebra*					2	2	I	11
Serrapinnus notomelas					1	2	A	12
Moenkhausia bonita					1	1	I	13
Acestrorhynchus lacustris					1	1	P	14
Aphyocharax dentatus						1	Z-I	15
Steindachnerina brevipinna						1	D-A	16
Pyrrhulina australis						1	I	17
Serrasalmus marginatus						1	P	18
