# Supplementary material for: Loss of Rare Fish Species from Tropical Floodplain Food Webs Affects Community Structure and Ecosystem Multifunctionality in a Mesocosm Experiment
Source: PLoS One. 2014 Jan 8;9(1):e84568. doi: 10.1371/journal.pone.0084568 (PMC3885587; doi:10.1371/journal.pone.0084568)
Supplement: Table S4 — Mixed model, repeated measures ANOVA for each response variable and associated covariate models with direct trophic interactions. When a significant treatment by time interaction was observed, SLICE effects are reported. Significant results in bold (α = 0.05). (PDF) [file pone.0084568.s006.pdf]

Table S4. Mixed model, repeated measures ANOVA for each response variable and associated covariate models with direct trophic interactions. When a significant treatment by time interaction was observed, SLICE effects are reported. Significant results in bold ($\alpha = 0.05$).

							treatment x time slices				
Model	Transformation	Effect	nDF	dDF	F	p	Time	nDF	dDF	F	p
N_1	log10	treatment	5	18	19.25	**<0.0001**	1	5	18	12.37	**<0.0001**
		time	3	18	12.54	**0.0001**	2	5	18	17.3	**<0.0001**
		treatment x time	15	18	4.34	**0.002**	3	5	18	24.43	**<0.0001**
		fish biomass	1	18	52.57	**<0.0001**	4	5	18	23.49	**<0.0001**
N_2	log10	treatment	5	18	11.65	**<0.0001**	1	5	18	1.72	0.1808
		time	3	18	37.44	**<0.0001**	2	5	18	8.05	**0.0004**
		treatment x time	15	18	6.4	**0.0002**	3	5	18	12.59	**<0.0001**
		phytoplankton	1	18	6.77	**0.018**	4	5	18	8.32	**0.0003**
N_3	log10	treatment	5	18	18.5	**<0.0001**	1	5	18	3.07	**0.0356**
		time	3	18	58.61	**<0.0001**	2	5	18	14.57	**<0.0001**
		treatment x time	15	18	7.39	**<0.0001**	3	5	18	15.93	**<0.0001**
							4	5	18	11.88	**<0.0001**
P_1	log10	treatment	5	18	25.02	**<0.0001**	1	5	18	14.21	**<0.0001**
		time	3	18	33.86	**<0.0001**	2	5	18	31.74	**<0.0001**
		treatment x time	15	18	10.76	**<0.0001**	3	5	18	16.94	**<0.0001**
		phytoplankton	1	18	7.05	**0.0161**	4	5	18	11.58	**<0.0001**
		zooplankton	1	18	8.46	**0.0094**					
P_2	log10	treatment	5	18	23.34	**<0.0001**	1	5	18	14.7	**<0.0001**
		time	3	18	31.19	**<0.0001**	2	5	18	30.66	**<0.0001**
		treatment x time	15	18	9.97	**<0.0001**	3	5	18	15.95	**<0.0001**
		phytoplankton	1	18	9.98	**0.0054**	4	5	18	11.31	**<0.0001**
P_3	log10	treatment	5	18	33.78	**<0.0001**	1	5	18	24.72	**<0.0001**
		time	3	18	38.09	**<0.0001**	2	5	18	47.77	**<0.0001**
		treatment x time	15	18	12.17	**<0.0001**	3	5	18	19.07	**<0.0001**
		zooplankton	1	18	11.45	**0.0033**	4	5	18	13.91	**<0.0001**
P_4	log10	treatment	5	18	12.62	**<0.0001**	1	5	18	5.41	**0.0033**
		time	3	18	24.36	**<0.0001**	2	5	18	7.82	**0.0005**
		treatment x time	15	18	7.36	**<0.0001**	3	5	18	17.26	**<0.0001**
		fish biomass	1	18	15.22	**0.001**	4	5	18	13.2	**<0.0001**
$phyto_1$	log10	treatment	5	18	16.92	**<0.0001**					
		time	3	18	11.4	**0.0002**					

		treatment x time	15	18	2.06	0.0734					
phyto$_2$	log10	treatment	5	18	4.13	**0.0112**					
		time	3	18	1.82	0.1804					
		treatment x time	15	18	1.7	0.1408					
		fish biomass	1	18	5.9	**0.0259**					
zoo$_1$	log10	treatment	5	18	12.17	**<0.0001**					
		time	3	18	13.91	**<0.0001**					
		treatment x time	15	18	0.089	0.5838					
		fish biomass	1	18	40.91	**<0.0001**					
BOM$_1$	none	treatment	5	18	1.56	0.2224					
		time	3	18	11.72	**0.0002**					
		treatment x time	15	18	0.87	0.6006					
BOM$_2$	none	treatment	5	18	1.82	0.1591					
		time	3	18	11.97	**0.0002**					
		treatment x time	15	18	0.87	0.6011					
		fish biomass	1	18	3.55	0.0756					
GPP$_1$	log10	treatment	5	18	30.22	**<0.0001**	1	5	18	5.62	**0.0027**
		time	3	18	37.69	**<0.0001**	2	5	18	16.04	**<0.0001**
		treatment x time	15	18	16.39	**<0.0001**	3	5	18	23.85	**<0.0001**
		fish biomass	1	18	29.34	**<0.0001**	4	5	18	52.3	**<0.0001**
GPP$_2$	log10	treatment	5	18	46.96	**<0.0001**	1	5	18	3.28	**0.0281**
		time	3	18	136.67	**<0.0001**	2	5	18	45.17	**<0.0001**
		treatment x time	15	18	31.61	**<0.0001**	3	5	18	23.97	**<0.0001**
							4	5	18	44.08	**<0.0001**
NPP$_1$	none	treatment	5	18	23.26	**<0.0001**	1	5	18	2.96	**0.0401**
		time	3	18	33.08	**<0.0001**	2	5	18	10.02	**0.0001**
		treatment x time	15	18	18.9	**<0.0001**	3	5	18	11.01	**<0.0001**
							4	5	18	19.07	**<0.0001**
NPP$_2$	none	treatment	5	18	6.42	**0.0014**	1	5	18	1.03	0.4313
		time	3	18	11.9	**0.0002**	2	5	18	3.59	**0.02**
		treatment x time	15	18	8.81	**<0.0001**	3	5	18	5	**0.0048**
		fish biomass	1	18	5.6	**0.0293**	4	5	18	14.69	**<0.0001**
