# Supplementary material for: Loss of Rare Fish Species from Tropical Floodplain Food Webs Affects Community Structure and Ecosystem Multifunctionality in a Mesocosm Experiment
Source: PLoS One. 2014 Jan 8;9(1):e84568. doi: 10.1371/journal.pone.0084568 (PMC3885587; doi:10.1371/journal.pone.0084568)
Supplement: Appendix S1 — List of species present from community sampling data from isolated lagoons in the Upper Paraná River floodplain (n = 11) during 2000–2007 austral springs. Species above the dashed line represent the pool of species (n = 18) used in experimental treatments. Eighteen species represent 82% of the total abundance and 47% of total biomass of individuals with mean SL <20 cm. (PDF) [file pone.0084568.s007.pdf]

Appendix S1. List of species present from community sampling data from isolated lagoons in the Upper Paraná River floodplain (n = 11) during 2000-2007 austral springs. Species above the dashed line represent the pool of species (n = 18) used in experimental treatments. Eighteen species represent 82% of the total abundance and 47% of total biomass of individuals with mean SL < 20 cm.

Species	Sum Rank Abundance	Total Abundance	Rank Biomass	Total Biomass (g)	Mean SL (cm)	Mean St Dev SL (cm)
Serrapinnus sp. 1*	1	1046[a]	20[a]	450[a]	2.26	0.24
Astyanax altiparanae	2	778	4	2695	4.24	1.45
Moenkhausia aff. *forestii*	3	623	19	498	2.73	0.50
Hyphessobrycon eques	4	420	28	199	2.40	0.38
Aphyocharax anisitsi	5	575	26	264	2.62	0.36
Steindachnerina insculpta	6	444	3	3217	5.52	2.18
Psellogrammus kennedyi‡	7	861[a]	8[a]	1626[a]	3.04	0.60
Loricariichthys platymetopon	8	129	6	2327	9.38	6.16
Roeboides descalvadensis	9	420	15	932	4.74	1.11
Hoplias aff. *malabaricus*	10	57	2	5938	14.70	7.18
Serrapinnus sp. 2¶	n/a	327	31	151	2.48	0.33
Characidium aff. *zebra*	11	82	39	37	2.69	0.64
*Serrapinnus notomelas**	12	123[a]	38[a]	42[a]	2.29	0.58
Moenkhausia bonita§	13	102[a]	36[a]	72[a]	2.65	0.34
Acestrorhynchus lacustris	14	54	9	1555	11.67	3.85
Aphyocharax dentatus†	15	161	34	100	2.91	0.78
Steindachnerina brevipinna	16	165	11	1480	5.95	2.33
Pyrrhulina australis	17	68	41	32	2.59	0.46
Serrasalmus marginatus	18	26	30	168	3.93	3.37
Cichlasoma paranaense	19	120	10	1545	5.17	2.18
Bryconamericus stramineus	20	219	25	286	4.13	0.63

Hyphessobrycon sp.	21	70	40	33	2.74	0.41
Pimelodella gracilis	22	85	24	328	6.38	1.74
Leporinus lacustris	23	32	22	377	5.72	3.09
Brachyhypopomus cf. *pinnicaudatus*	24	43	33	104	8.43	2.42
Gymnotus spp.	25	17	18	565	16.66	7.03
Cyphocharax nagellii	26	12	37	63	4.62	1.81
Hoplosternum littoral	27	23	5	2344	14.01	2.23
Crenicichla britskii	28	5	46	16	4.58	1.69
Leporinus obtusidens	29	6	14	1200	15.60	8.91
Laetacara sp.	30	55	32	127	3.51	0.81
Aphyocharax sp 1†	31	14	53	4	2.43	0.33
Hoplerythrinus unitaeniatus	32	6	16	921	18.33	4.04
Satanoperca pappaterra	33	35	45	16	1.92	0.45
Astronotus crassipinnis	34	4	7	1991	20.55	2.44
Synbranchus marmoratus	35	3	51	8	---	0.00
Apareidon affinis	36	3	56	2	3.10	0.95
Characidium sp.	37	3	57	1	2.37	0.76
Catathyridium jenynsii	38	3	27	218	7.33	7.94
Pamphorichthys sp.	39	5	58	1	1.90	0.57
Prochilodus lineatus	40	189	1	15106	13.09	2.15
Eigenmannia trilineata	41	4	43	24	12.75	3.15
Sternopygus macrurus	42	2	54	2	6.90	0.85
Odontostilbe sp.	43	6	55	2	2.15	0.64
Astyanax schubarti	44	2	44	17	6.40	0.85
Hoplias sp. 2	45	1	29	183	21.00	---
Potamotrygon cf. *motoro*	46	2	13	1303	22.00	1.41
Astyanax aff. *fasciatus*	47	2	50	9	7.60	0.00
Myloplus tiete	48	1	52	6	5.00	---

Pterygoplichthys anisitsi	49	1	17	677	32.20	---
Serrasalmus maculatus	50	1	23	328	19.60	---
Cyphocharax modestus	51	2	42	32	7.35	0.21
Pseudoplatystoma corruscans	52	3	12	1384	32.37	9.02
Parauchenipterus galeatus	53	1	48	11	6.70	---
Hoplias sp. 1	54	1	21	448	30.00	---
Pimelodella avanhandavae	55	1	49	9	9.00	---
Salminus brasiliensis	56	1	---	---	---	0.00
Metynnis lippincottianus	57	2	47	15	5.50	0.42
Hypostomus ancistroides	58	1	35	99	15.00	---

Nomenclature follows Graca, W. J. and C. S. Pavanelli, editors. 2007. Peixes da planície de inundação do alto rio Paraná e áreas adjacentes. Editora do Universidade Estadual de Maringá, Maringá, Brazil

* Taxonomic ambiguity among *Serrapinnus* species early in the data set likely caused misidentification between *Serrapinnus notomelas* and *Serrapinnus* sp. 1 Therefore, species exchanged positions based on current observed abundances in isolated lagoons

† Taxonomic ambiguity among *Aphyocharax* species early in the data set likely caused misidentification between *Aphyocharax dentatus* and *Aphyocharax* sp. 1 Therefore, species exchanged positions based on current observed abundances in isolated lagoons

‡ Replaced *Moenkhausia* aff. *intermedia* due to captive sensitivity of this species. *Psellogrammus kennedyi* is similar in ecology and size and currently found in and abundances similar to *Moenkhausia* aff. *intermedia*

§ Replaced *Hemigrammus marginatus* due to captive sensitivity of this species. *Moenkhausia bonita* is similar in ecology and size and currently found in and abundances similar to *Hemigrammus marginatus*

¶ Species removed for no longer found in isolated lagoons

[a] Value is reflective of species it replaced

--- No information
